# The role of matrix metalloproteinase 9 in immune-mediated skin diseases

**DOI:** 10.3389/fimmu.2026.1802353

**Published:** 2026-03-23

**Authors:** Ke Xu, Min Li, Fengming Hu, Jian Gong, Fangrong Liu, Qiao Liu, Weiwei Wu

**Affiliations:** 1 Clinical School of Medicine, Jiangxi University of Traditional Chinese Medicine, Nanchang, Jiangxi, China; 2 Department of Dermatology, The Fifth People’s Hospital of Hainan Province, Haikou, Hainan, China; 3 Department of Dermatology, Affliated Dermatology Hospital of Hainan Medical University, Haikou, Hainan, China; 4 Dermatology Hospital of Jiangxi Province, Nanchang, Jiangxi, China; 5 Jiangxi Provincial Clinical Research Center for Skin Diseases, Nanchang, Jiangxi, China

**Keywords:** angiogenesis, biomarker, dermatological disorders, immune-mediated diseases, inflammatory response, matrix metalloproteinase-9, therapeutic target, tissue remodeling

## Abstract

MMP-9, dependent on zinc, is a key endopeptidase involved in tissue homeostasis, inflammation, and immune-related pathological processes through its role in extracellular matrix degradation and remodeling. Compared with other immune-associated skin diseases, compelling evidence indicates aberrant MMP-9 expression in psoriasis, vitiligo, bullous pemphigoid, and melanoma. MMP-9 modulates the pathological progression of these disorders through multiple mechanisms, including regulation of immune responses, inflammatory cascades, angiogenesis, and tissue remodeling, thereby demonstrating considerable translational potential. MMP-9 emerges as a promising biomarker and therapeutic target, but clinical validation remains limited. Currently, the clinical application of MMP-9 inhibitors is plagued by several critical drawbacks, such as poor selectivity, off-target effects, severe toxic side effects, and unsatisfactory therapeutic efficacy. Therefore, the exploration of novel MMP-9 inhibitors and the conduction of well-designed, adequately powered clinical trials are urgently warranted and of great clinical necessity. This comprehensive review systematically examines the molecular regulatory network of MMP-9 in immune-mediated dermatological disorders and evaluates its translational capacity as a biomarker and therapeutic target, along with its clinical applications and key challenges.

## 1 Introduction

The family of matrix metalloproteinases (MMPs) comprises multi-domain, zinc-dependent enzymes that modulate critical biological processes, including embryonic development, tissue regeneration, chronic inflammation, and tumorigenesis, by precisely regulating extracellular matrix (ECM) degradation and remodeling (1, 2). MMP-9, a prominent secreted isoform within the matrix metalloproteinase family, plays a critical and indispensable role in wound healing, immune regulation, and cutaneous development (3). In the last several years, aberrant expression of MMP-9 and its underlying regulatory mechanisms in immune-mediated dermatological disorders, including psoriasis, vitiligo, bullous pemphigoid, and melanoma, have emerged as a research hotspot in the interdisciplinary fields of dermatology and molecular immunology, yet the precise molecular mechanisms remain largely elusive. Although a number of reviews have addressed the biological functions of the matrix metalloproteinase family or the pathogenesis of individual dermatological disorders, existing studies still have certain limitations and a narrow research focus: some reviews only concentrate on the overall roles of the MMP family and lack in-depth elaboration on the specific regulatory mechanisms of MMP-9, a key therapeutic target, while others are confined to investigating the mechanisms of MMP-9 in a single dermatological disorder, with no comprehensive review yet systematically elucidating the multifaceted roles of MMP-9 in dermatological disorders and its translational potential for targeted therapy.

In this review, we performed a comprehensive literature search in the PubMed, Web of Science, and Embase databases for publications from 2015 to 2025 using the search strategy: (matrix metalloproteinase 9 OR MMP-9 OR gelatinase B) AND (dermatosis OR skin disease OR cutaneous disease) AND (immune-mediated OR inflammatory OR autoimmune). Following the application of strict inclusion and exclusion criteria, a total of 88 eligible articles were finally identified. Among these, the majority of publications with high levels of evidentiary support focused on psoriasis, vitiligo, bullous pemphigoid, and melanoma, which therefore constitute the core focus of this review.

This review comprehensively elucidates the core pathological mechanisms mediated by MMP-9 in skin diseases, such as immune cell infiltration and extracellular matrix (ECM) remodeling, and further correlates these mechanisms with emerging therapeutic strategies, including MMP-9 inhibitor development, hydrogel-based targeted drug delivery, and natural product-mediated intervention, aiming to achieve an integrated analysis of immune regulatory mechanisms and therapeutic platforms for immune-mediated dermatological disorders. Furthermore, we delve into the clinical potential of MMP-9 as a diagnostic and prognostic biomarker, as well as the current research bottlenecks and future directions in this field, with the ultimate aim of providing novel insights and theoretical guidance for investigating the pathogenesis of immune-mediated dermatological disorders and formulating precise targeted therapeutic approaches.

## 2 Structure and function of MMP-9

### 2.1 Structural architecture of MMP-9

MMP-9 is a key member of the gelatinase subfamily within the matrix metalloproteinase (MMP) family. The enzyme degrades a range of skin-relevant substrates, including type IV and V collagen, laminin, elastin, and gelatin, as well as other key extracellular matrix proteins. MMP-9 is genetically mapped to the human chromosomal region 20q11.1 to 20q13.1, which harbors gene clusters associated with extracellular matrix remodeling, inflammatory responses, and tumor metastasis. This chromosomal localization suggests a coordinated regulatory function for MMP-9 in diverse pathophysiological processes (4).

MMP-9 has 707 amino acids. Its multiple domains provide substrate specificity and functional versatility. The protein architecture includes an N-terminal signal peptide, propeptide domain, catalytic domain, fibronectin type II repeats, a hinge region, and a hemopexin-like domain. Each domain’s function is directly linked to skin physiological and pathological processes. The N-terminal signal peptide is essential for MMP-9 secretion from skin-derived cells. It guides MMP-9 zymogen processing through the endoplasmic reticulum-Golgi apparatus in keratinocytes, dermal neutrophils, and fibroblasts. This step is required for the local synthesis and release of MMP-9 in the skin. The propeptide domain maintains enzymatic latency via the cysteine-switch mechanism (Cys^99^-Zn²^+^ coordination). This inhibition prevents premature activation of epidermal and immune cells in the skin. Such control is critical to avoid unintended ECM degradation, compromised barrier integrity, and nonspecific inflammation.

As the core functional domain responsible for skin ECM degradation, the catalytic domain specifically hydrolyzes substrates including denatured collagen and basement membrane type IV collagen, thereby directly participating in skin tissue remodeling, inflammatory cell migration across the basement membrane, and tumor cell invasion. The fibronectin type II repeats constitute the core region of MMP-9 for recognizing skin substrates. Formed by hydrophobic amino acids and conserved cysteines into a characteristic hydrophobic binding pocket, they specifically bind key basement membrane substrates, such as type IV collagen and elastin. A subsequent conformational change brings the catalytic domain closer to the skin substrate, initiating hydrolysis. Furthermore, they synergistically enhance binding affinity and targeting toward skin ECM substrates. Additionally, these repeats participate in adhesive interactions between melanocytes and the basement membrane of the skin. The hinge region provides the structural basis for MMP-9’s interaction with skin cell surfaces, conferring flexibility that enables efficient binding to receptors on keratinocytes and dermal vascular endothelial cells, as well as to ECM components. This region is structurally crucial for MMP-9’s role in recruiting inflammatory cells and promoting angiogenesis in the skin. The hemopexin-like domain directly regulates the local activity and immune functions of MMP-9 in the skin by specifically binding to tissue inhibitors of metalloproteinases (TIMPs) to modulate its enzymatic activity, while also participating in interactions with adhesion receptors on melanocytes and signaling receptors on immune cells, thereby mediating the activation and signal transduction of local immune cells in the skin (5). This multi-domain architecture endows MMP-9 with the capacity to selectively degrade type IV collagen, gelatin, laminin, and other ECM components, thereby modulating basement membrane integrity and cellular migration processes (6, 7).

**Figure 1:**
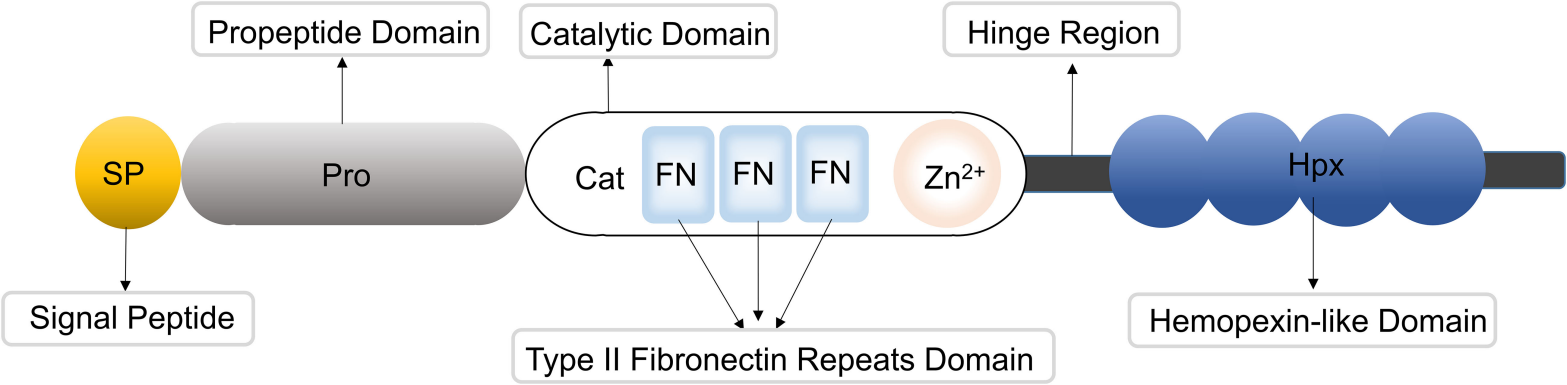
MMP-9 encompasses an N-terminal signal peptide, a propeptide domain, a catalytic domain, fibronectin type II repeats, a hinge region, and a hemopexin-like domain.

The structure of MMP-9 is shown in **Figure 1**.

### 2.2 Functional roles of MMP-9

#### 2.2.1 Promotion of tissue remodeling and angiogenesis

In normal skin architecture, keratinocytes constitute the predominant cellular component of the epidermis, whereas the dermis is primarily composed of extracellular matrix and fibroblasts.

Laminin and type I and type II collagen represent the principal constituents of the ECM. MMP-9 is secreted by various cell types, including keratinocytes and fibroblasts, and orchestrates diverse pathophysiological processes, such as cellular proliferation, migration, differentiation, and angiogenesis, through ECM degradation (8). Studies have demonstrated that MMP-9 liberates ECM-sequestered vascular endothelial growth factor (VEGF), thereby promoting endothelial cell migration and lumen formation, which provide essential nutritional and oxygen support for tissue repair (9). MMP-9 further promotes neovascularization by degrading collagen fibers and fibrinogen to create a conducive growth environment (10). This characteristic enables MMP-9 to participate in skin diseases characterized primarily by vascular hyperplasia. Moreover, elevated MMP-9 expression may impair melanocyte viability and proliferation, ultimately contributing to the pathogenesis of vitiligo (11).

MMP-9 plays a crucial role in skin tissue remodeling and the regulation of angiogenesis, which is essential for maintaining skin homeostasis, and is also involved in the growth and metastasis of various malignancies. Zhang et al. (12) identified that MMP-9 serves as a key mediator in Acinetobacter spp., zinc oxide-induced acne-associated skin barrier dysfunction, and delayed wound healing. Animal studies have demonstrated that radiofrequency treatment can downregulate MMP-9 expression by modulating macrophage and fibroblast activation, thereby preventing the upregulation of MMP-9 after UV irradiation-induced skin photoaging and protecting dermal collagen and elastin (13). It is noteworthy that MMP-9 is significantly overexpressed in various malignant tumors, including colorectal cancer, breast cancer, and melanoma. By disrupting intercellular ECM adhesion and promoting tumor angiogenesis, MMP-9 accelerates tumor proliferation and invasion (14–17).

#### 2.2.2 Involvement in inflammatory response and immune process

MMP-9 is localized in the cytoplasm of leukocytes and macrophages. By cleaving and inactivating the α subunit of 5′-adenosine monophosphate-activated protein kinase (AMPK), it regulates cellular energy homeostasis, thereby influencing innate immunity and inflammatory responses. Research has shown that MMP-9 activates Bruton’s tyrosine kinase (BTK) in both mouse and human neutrophils, thereby mediating NLRP3 inflammasome secretion and enhancing inflammation by regulating neutrophil extracellular trap (NET) release (18). Under inflammatory conditions, MMP-9 expression is markedly upregulated, facilitating the migration and infiltration of inflammatory cells into the epidermis through ECM disruption and pathway formation (8). FAN et al. (19) showed that CXCL2-mediated activation of endogenous MMP-9 expression in neutrophils promotes basement membrane degradation, leading to excessive transepidermal migration and epidermal accumulation of neutrophils, thereby contributing to inflammation in skin diseases.

Matthew et al. (20) reported that MMP-9 contributes to the neuroinflammatory response by inducing neuronal and glial cell damage, accelerating the release of pro-inflammatory factors, and disrupting neuron-glial interactions. During sepsis, MMP-9 drives an inflammatory amplification cycle by promoting leukocyte overactivation and proliferation, inducing cytokine/chemokine release, and further activating immune cells (18). Additionally, MMP-9 initiates vitiligo inflammatory cascades by cleaving soluble stem cell factor (SCF) during early disease development, thereby recruiting mast cells to inflamed skin regions (21). Furthermore, MMP-9 influences immune cell activity and differentiation via activation of the TGF-β signaling pathway and participates in fibroblast-mediated tissue remodeling (22). Notably, MMP-9 is a core factor involved in the inflammatory response of atherosclerosis. Macrophage-derived MMP-9 can disrupt the integrity of the intima by hydrolyzing the vascular wall extracellular matrix, promote the infiltration of monocytes/macrophages into lesions and lipid accumulation, and shape a local pro-inflammatory microenvironment. It can also promote endothelial-to-mesenchymal transition (EndMT) by activating the TGF-β and Notch signaling pathways. EndMT forms a positive feedback loop with inflammatory signals, which can further amplify vascular inflammation and exacerbate atherosclerosis (23, 24). Inhibition of MMP-9 can reduce the level of C-reactive protein (CRP) in aortic atherosclerotic plaques, decrease local inflammatory responses, and maintain plaque stability (25).

MMP-9 displays context-dependent functional heterogeneity across disease microenvironments. In inflammatory skin and systemic diseases, it acts as a pro-inflammatory factor, amplifying inflammation and tissue damage by degrading ECM and activating inflammatory cascades. Conversely, in malignancies such as melanoma, MMP-9 shifts toward an anti-inflammatory and immunosuppressive phenotype. Through its proteolytic activity, it cleaves activation receptors on antitumor immune cells, thereby suppressing immune activation and cytotoxicity. This divergence underpins its opposing roles in inflammatory versus neoplastic pathologies (26).

**Figure 2:**
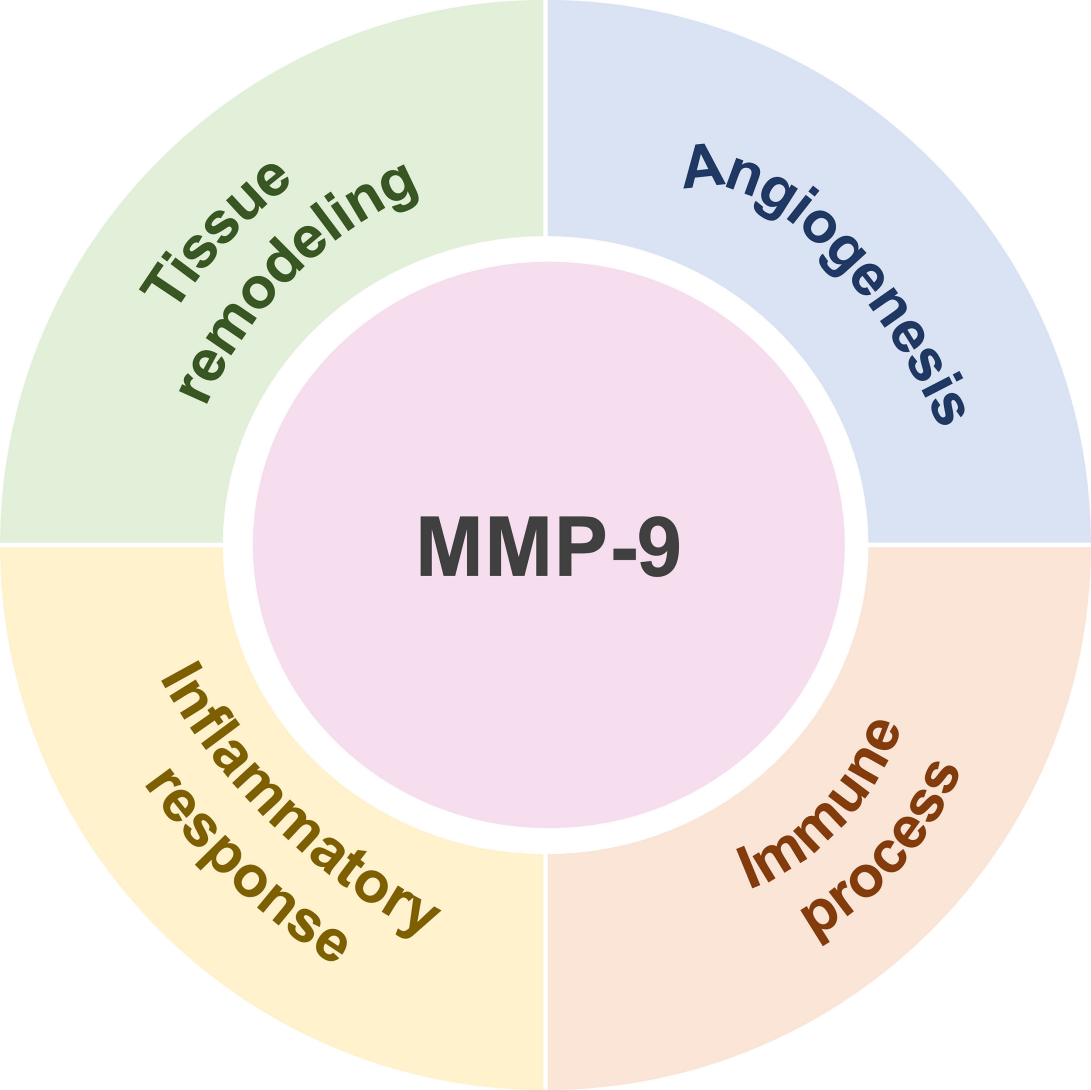
MMP-9 can promote tissue remodeling and angiogenesis, as well as participate in controlling the activity of immunological processes and inflammatory reactions.

The functions of MMP-9 are shown in **Figure 2**.

## 3 MMP-9 and immune-mediated skin diseases

### 3.1 MMP-9 and psoriasis

Psoriasis is a chronic, relapsing, inflammatory skin disease characterized clinically by scaly erythematous plaques, affecting over 125 million people worldwide (27). The recurrent nature of the disease necessitates long-term treatment and lifelong disease management, imposing a significant psychological burden and substantial lifestyle impact on patients and their families (28). The etiology of psoriasis is complex, involving interactions between immunity and genetics. Its major pathological features include hyperproliferation and abnormal differentiation of keratinocytes, inflammatory cell infiltration, and pathological angiogenesis (29).

MMP-9 is critically implicated in the pathogenesis of psoriasis, demonstrating dysregulated expression both locally in lesional skin and systemically. Clinical analyses reveal significantly elevated MMP-9 levels in psoriatic plaques compared with healthy skin. Receiver operating characteristic (ROC) curve analysis yields an area under the curve (AUC) of 0.77, indicating promising diagnostic potential (30). Correspondingly, increased MMP-9 concentrations in peripheral blood and heightened expression in monocytes further corroborate its role in disease development (31). Wagner et al. (32) reported markedly elevated MMP-9 protein levels not only in lesional but also in non-lesional skin compared to controls, suggesting that its dysregulation is a systemic feature rather than confined to plaques. This supports the potential of MMP-9 as a biomarker even during clinically silent phases of psoriasis. However, as current evidence predominantly stems from correlative studies, large-scale prospective validation is required before it can be translated into clinical practice.

MMP-9 plays a role in the pathogenesis of psoriasis by regulating cellular signaling and enhancing keratinocyte migration. Furthermore, altered MMP-9 expression in psoriatic skin promotes neovascularization. MMP-9-mediated tissue remodeling affects immune cell activation and modulates psoriatic epidermal inflammatory responses by regulating lymphocyte and neutrophil migration (33, 34). *In vitro* studies indicate that interleukin-17 (IL-17) and tumor necrosis factor-α (TNF-α) regulate MMP-9 secretion from monocytes, thereby modulating tissue remodeling in psoriatic lesions (35, 36).

Elevated MMP-9 levels may impair skin re-epithelialization. In psoriasis, its overexpression can further disrupt ECM integrity, impede tissue remodeling, sustain the inflammatory microenvironment, and promote abnormal keratinocyte proliferation (37). The study by Chen et al. (38) revealed that MMP-9 is expressed within resting human neutrophils isolated from psoriatic lesions. *In vitro* experiments demonstrated that upon TNF-α stimulation, neutrophils release active MMP-9. As neutrophils migrate from the vasculature into the dermis, MMP-9 can activate the ERK-1/2 and p38/MAPK signaling pathways in vascular endothelial cells. This leads to vasodilation and increased vascular permeability, thereby accelerating pathological angiogenesis. Furthermore, MMP-9 promotes endothelial secretion of ICAM-1, IL-1β, and CXCL1, thereby enhancing the migratory capacity of CD4^+^ T cells and facilitating their infiltration from blood vessels into psoriatic lesions. The therapeutic potential of targeting MMP-9 was validated in animal studies, where an MMP-9 inhibitor significantly alleviated skin lesions and vascular dilation in imiquimod- or IL-23-induced mouse models of psoriasis (39).

MMP-9 levels are positively correlated with psoriasis severity. Chihaoui et al. (40) compared serum MMP-9 levels between patients with psoriasis and healthy controls; logistic regression analysis identified MMP-9 as an independent risk factor for the disease. In psoriatic lesions, the inflammatory milieu together with elevated VEGF promotes MMP-9 secretion from T cells, neutrophils, fibroblasts, macrophages, keratinocytes, and vascular endothelial cells. MMP-9 contributes to angiogenesis and activates endothelial cells by facilitating the degradation of the vascular basement membrane and the pericellular extracellular matrix. Elevated serum MMP-9 levels in patients with psoriasis reflect extensive tissue damage and heightened inflammatory activity (40). Bharatha et al. (41) compared the concentrations of MMP-9 in healthy human biopsy specimens and neutrophils stimulated by IMQ with those stimulated by BQ, using Western blot and gelatin gel zymography to analyze the protein level and activity of MMP-9 in IMQ-stimulated neutrophils alongside BD1- and UCB-treated neutrophils from healthy human tissue sources. Results demonstrated that IMQ-mediated neutrophil activation increases MMP-9 levels. Subsequent animal studies confirmed that BD1 and UCB inhibited MMP-9 activity and protein expression while suppressing neutrophil-specific chemokines and pro-inflammatory cytokines in psoriasis-like murine skin, consequently ameliorating IMQ-induced cutaneous inflammation. The data indicate that anti-MMP-9 therapy may represent a promising therapeutic approach for psoriasis treatment.

In summary, current evidence indicates that MMP-9 is a key biomarker in the pathogenesis of psoriasis, with a positive correlation with disease severity. It promotes psoriatic inflammation, angiogenesis, and epidermal hyperproliferation by activating vascular endothelial cells, recruiting inflammatory cells, and modulating keratinocyte proliferation. Although anti-MMP-9 therapies have shown promising results in animal studies, corresponding clinical trials remain lacking. Further clinical research is needed to establish the safety and efficacy of MMP-9-targeted treatments for psoriasis.

**Figure 3:**
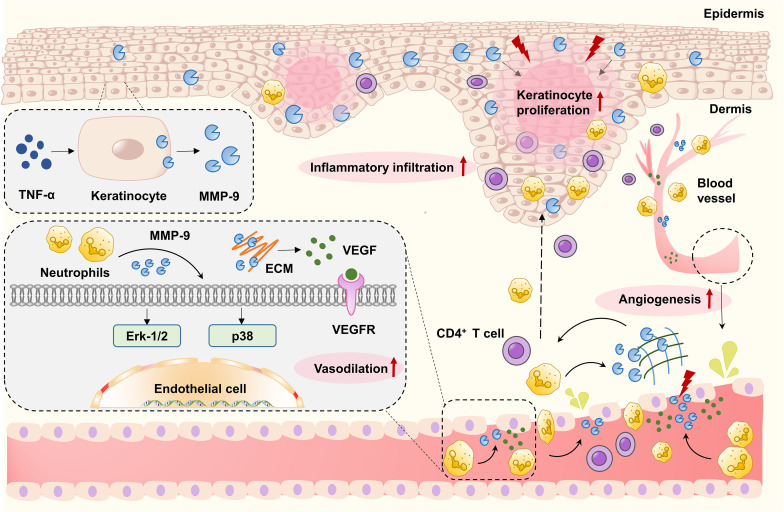
In psoriasis, MMP-9-mediated tissue remodeling can affect immune cell activation and regulate psoriasis epidermal inflammatory response by modulating lymphocyte and neutrophil migration. The elevation of MMP-9 in psoriasis plaques can disrupt the ECM integrity and facilitate the abnormal proliferation of keratinocytes. In dermal blood vessels, MMP-9 derived from neutrophils encourages the development of psoriasis lesions by activating skin endothelial cells, leading to vasodilation and increased vascular permeability. MMP-9 can promote the release of VEGF by explaining the vascular basement membrane and endothelial ECM, and induce vasodilation and neovascularization by activating the ERK1/2 and p38 MAPK pathways in skin endothelial cells.

**Figure 3** depicts the function of MMP-9 in psoriasis.

### 3.2 MMP-9 and vitiligo

Vitiligo is an autoimmune, pigment-loss dermatosis with a global prevalence of approximately 0.1%-2% (42). It can occur at any age, is more common in children and adolescents, shows no significant gender predominance, and typically follows a chronic, progressive course (43). The core clinical manifestation is well-demarcated white patches on the skin resulting from melanocyte apoptosis and dysfunction, which may involve the entire skin surface and mucous membranes. The disease is clinically classified into segmental and non-segmental types, with the latter accounting for approximately 80% of cases (44). Some patients may have associated autoimmune disorders such as thyroid disease and diabetes. As a classic disfiguring dermatological condition, vitiligo not only affects the patient’s appearance but also readily leads to psychological issues, including social avoidance, anxiety, and depression, significantly impairing mental health and quality of life (45). Research indicates that pigmentation in vitiligo necessitates the migration of melanocytes from the usually pigmented margins of vitiligo lesions into the depigmented epidermis, a process dependent on extracellular matrix remodeling and cell movement, and governed by the transcriptional activation of MMP-9 (46).

Samar et al. (47) conducted a clinical case-control study and observed significantly lower MMP-9 expression in the basal cell layer, hair follicle epithelium, and dermal matrix of vitiligo lesions compared to non-lesional skin and healthy controls. They proposed that reduced MMP-9 expression in lesional tissue may impair melanoblast migration and thereby hinder melanin regeneration. This study supports the role of tissue MMP-9 as an important predictor of vitiligo severity. Another investigation confirmed that patients with stable non-segmental vitiligo (NSV) exhibit lower systemic levels of MMP-9, suggesting that vitiligo pathogenesis is associated with an imbalance in MMP-9. Direct targeting of MMP-9 may therefore represent a potential therapeutic strategy for vitiligo (48).

Notably, in early-stage vitiligo, keratinocytes can upregulate MMP-9 expression in response to oxidative stress or external stimuli, thereby contributing to disease onset (49). Current studies have shown that in vitiligo, keratinocytes can produce two variants of stem cell factor (SCF) through alternative splicing: full-length SCF (fSCF) and membrane-bound SCF (mSCF). MMP-9 cleaves fSCF to generate soluble SCF (sSCF), which provokes and mobilizes skin mast cells (21). Further studies have confirmed that, in vitiligo, mast cell recruitment to inflamed skin sites is driven by soluble stem cell factors released by keratinocytes through stress-enhanced MMP-9-dependent proteolysis (21).

Another study demonstrated that house dust mites can induce innate immune and pro-inflammatory Th1/Th2 immune responses, disrupt adherens junctions in both normal and vitiligo skin, and may lead to leukoderma or melanocyte loss in non-lesional areas of vitiligo (50). Further *in vitro* experiments confirmed that house dust mites upregulate MMP-9 expression in vitiligo keratinocytes and degrade E-cadherin, resulting in melanocyte loss. The selective MMP-9 inhibitor Ab142180 was shown to restore E-cadherin expression and suppress house dust mite-induced melanocyte depletion. These findings support the role of house dust mites as an exogenous triggering factor in vitiligo pathogenesis and suggest that topical MMP-9 inhibitors may represent a viable therapeutic strategy. Moreover, MMP-9 is involved in UVB-induced melanocyte migration, and its expression is tightly regulated by the p53-TRPM1/miR-211-MMP-9 signaling axis, revealing a novel molecular pathway for therapeutic intervention in vitiligo (51).

Boukhedouni et al. (52) demonstrated that compared to healthy individuals and patients with stable vitiligo, those with active disease exhibit significantly elevated MMP-9 expression in both lesional skin and serum. Transcriptomic analysis of peri-lesional skin revealed MMP-9 among the top 10 upregulated genes in active vitiligo, highlighting its role in regulating prominent inflammatory pathways. Further investigation using a reconstructed pigmented epidermal 3D model confirmed that keratinocytes, when stimulated by IFN-γ and TNF-α, robustly produce MMP-9. This elevated MMP-9 expression promotes melanocyte apoptosis in the epidermal basal layer by disrupting E-cadherin. Subsequent animal studies showed that treatment with the gelatinase inhibitor SB-3CT or the selective MMP-9 inhibitor (ab142180) could reverse the significant melanocyte loss associated with E-cadherin disruption following IFN-γ and TNF-α exposure. These findings indicate that inhibiting MMP-9 correlates with enhanced stability of epidermal melanocytes, further substantiating MMP-9’s involvement in melanocyte loss.

A new investigation reveals that adhesion defects may cause depigmentation in vitiligo. In melanocytes from individuals with vitiligo, silencing of the Ets-1 transcription factor is associated with reduced MMP-9 activity. The persistent low expression of MMP-9 may lead to shedding of epithelial cadherin (E-cadherin), which, in turn, hinders melanocyte migration from the edge of the lesion to the depigmented area and impairs the skin’s repigmentation process (53, 54). Srivastava et al. (46) further confirmed, through clinical experiments, that ETS-1 expression is deficient in the cutaneous tissues of vitiligo patients. Meanwhile, the expression of MMP-2 and MMP-9, as well as that of cell adhesion molecules (E-cadherin, ICAM-1, and ITGA-1), is significantly reduced. After silencing ETS-1 with siRNA, the expression of adhesion molecules, including E-cadherin, ICAM-1, ITGA-1, and MMP-9, in normal melanocytes also decreased significantly, confirming that ETS-1 deficiency can directly affect MMP-9 and related adhesion factors.

These findings suggest that MMP-9 may serve as a potential biomarker for staging and targeted therapy in vitiligo. In patients with stable vitiligo, silencing of the melanocyte Ets-1 transcription factor reduces the expression of MMP-9, E-cadherin, ICAM-1, and ITGA-1, thereby impeding melanocyte migration and pigment regeneration. In contrast, during the active phase, IFN-γ and TNF-α stimulate keratinocytes to produce MMP-9, which subsequently mediates E-cadherin degradation and promotes melanocyte apoptosis. Notably, MMP-9 exhibits differential expression between keratinocytes and melanocytes, likely influenced by cell type and disease stage. This observation underscores the need to consider the stage-specific and cell-type-dependent pathogenic roles of MMP-9 in the clinical development and application of MMP-9 inhibitors. Determining how to select appropriate treatment regimens based on disease course warrants further in-depth clinical investigation.

**Figure 4:**
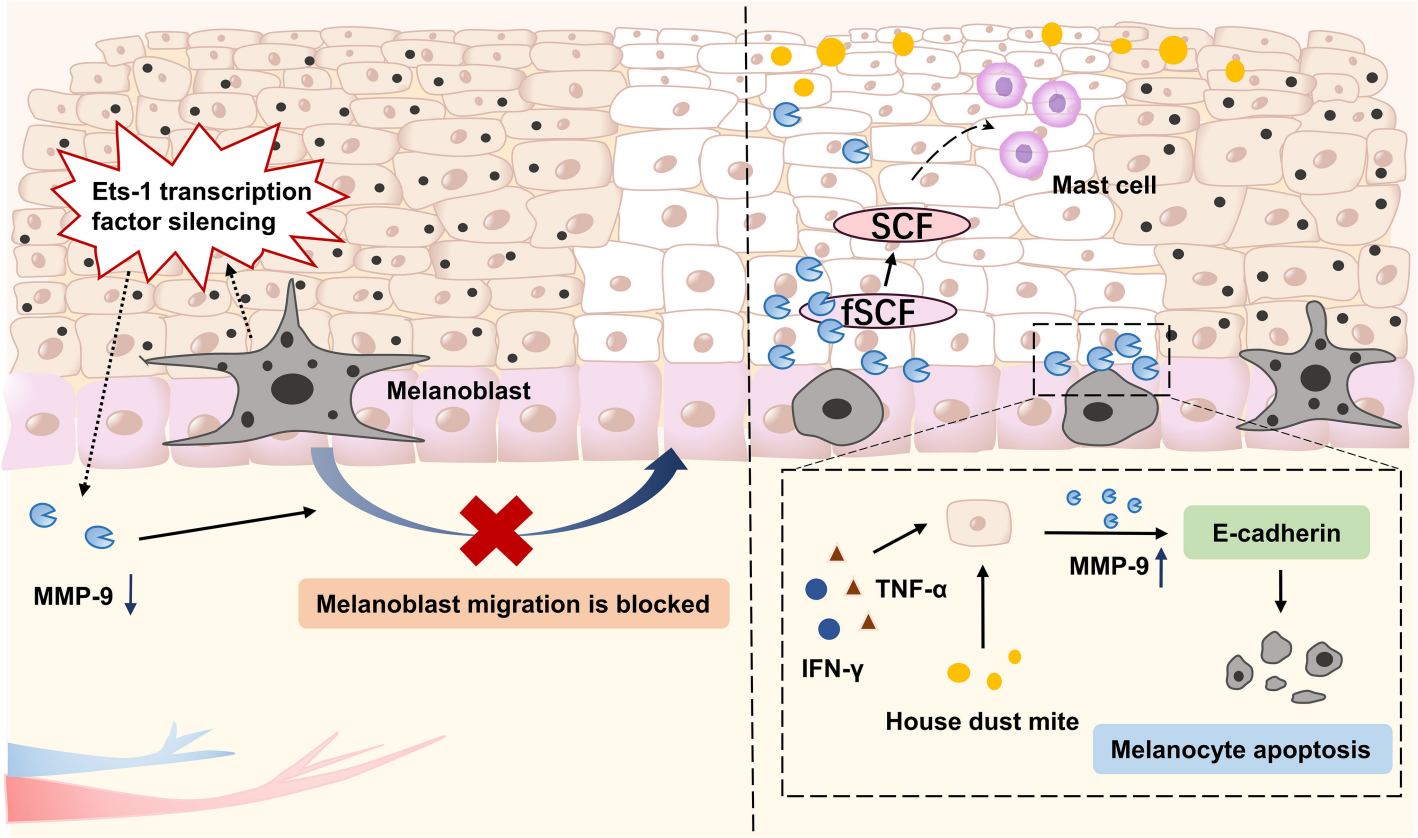
Mechanistic role of MMP-9 in vitiligo pathogenesis. In vitiligo lesions, silencing of the Ets-1 transcription factor in melanocytes significantly reduces the activity and expression of MMP-9, thereby impairing the migration of melanocytes from the periphery of the lesion to the pigmented area and hindering the re-deposition of skin pigment. Meanwhile, exogenous house dust mite, IFN-γ, and TNF-α can stimulate keratinocytes to secrete MMP-9, which degrades E-cadherin, disrupts the adhesion between melanocytes and the basement membrane, and thus induces melanocyte apoptosis. In addition, MMP-9 can also produce sSCF by cleaving SCF, thereby stimulating and recruiting skin mast cells and exacerbating the inflammatory response in vitiligo.

The role of MMP-9 in vitiligo is seen in **Figure 4**.

### 3.3 MMP-9 and bullous pemphigoid

Bullous pemphigoid (BP) is a prevalent autoimmune blistering disorder that primarily affects the elderly. Clinically, it is characterized by the appearance of clear, tense vesicles and bullae of variable size on either normal or erythematous skin, with a negative Nikolsky sign. The majority of patients present with pruritus of varying intensity, significantly compromising their quality of life. Pathologically, the disease is characterized by a prominent eosinophilic inflammatory infiltrate and the production of IgG and IgE autoantibodies targeting the BP180 and BP230 antigens within the basement membrane zone (55). MMP-9 concentrations are markedly increased in both serum and lesional skin tissues of BP patients, identifying MMP-9 as a crucial biomarker. Neutrophils and eosinophils represent the primary cellular sources of MMP-9 in BP (56–58).

The 16th non-collagenous domain (NC16A) of the BP180 extracellular domain represents the principal antigenic target for IgG antibodies, with anti-NC16A IgG autoantibodies identified in over 90% of patients with BP (59, 60). Prior research has demonstrated that MMP-9 can degrade the extracellular domain of BP180 *in vitro*, but the direct mechanism of action *in vivo* remains to be elucidated (61, 62). Shimanovich et al. (63) showed that blocking MMP-9 activity prevented blister formation in an IgG-mediated ex vivo human skin model of BP, suggesting that MMP-9-mediated neutrophil activation is closely associated with the dermal-epidermal separation process. Furthermore, studies have shown that anti-NC16A IgE/NC16A immune complexes can trigger MMP-9 secretion from eosinophils, and MMP-9-deficient mice exhibit partial resistance to BP-like lesions induced by anti-NC16A IgE. These results underscore MMP-9’s pivotal function in orchestrating eosinophil recruitment and mediating subsequent tissue injury (64).

As indicated above, in bullous pemphigoid, MMP-9 released by neutrophils and eosinophils promotes blister formation and epidermal-dermal separation by mediating inflammatory responses and tissue damage. Consequently, modulating MMP-9 activity may represent a potential therapeutic target for interrupting this pathological cascade.

**Figure 5:**
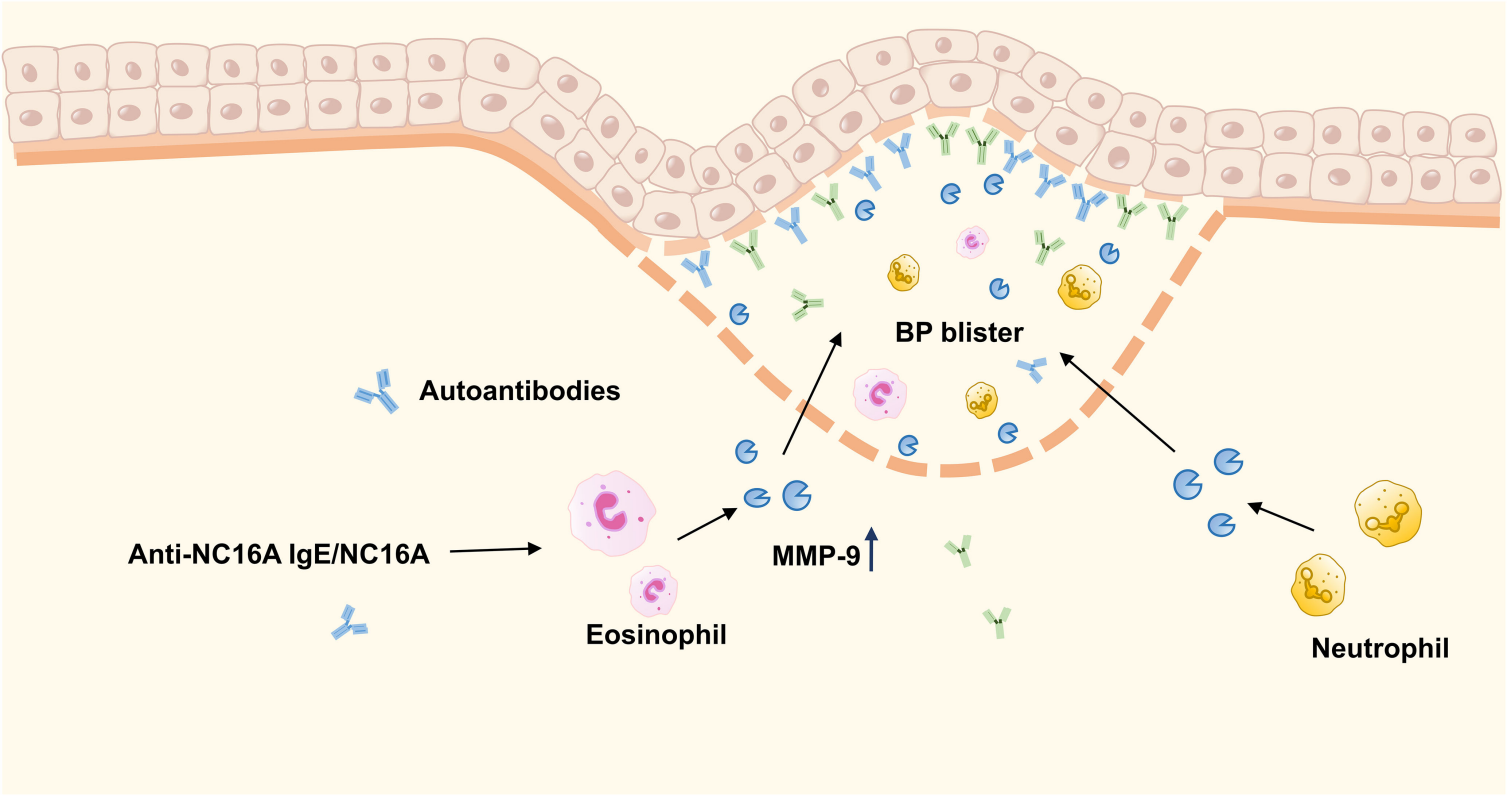
MMP-9 functional mechanism in Bullous Pemphigoid. In BP, MMP-9 released by neutrophils and eosinophils promotes blister formation and epidermal-dermal separation by mediating inflammatory responses and tissue damage processes. Additionally, MMP-9 derived from eosinophils is also associated with the formation of anti-NC16A IgE/NC16A immune complexes.

**Figure 5** illustrates the specific role of MMP-9 in bullous pemphigoid.

### 3.4 MMP-9 and melanoma

Melanoma (MM) is a malignant tumor originating from melanocytes in the skin, characterized by high invasiveness and metastatic potential. It most commonly arises in the skin but can also occur in mucosal surfaces and internal organs. Accounting for approximately 3% of all cancers, melanoma represents the most lethal form of skin cancer (65). MMP-9 serves as a key diagnostic and prognostic biomarker for melanoma, playing multiple roles in tumor growth, invasion, and metastasis through ECM degradation, neovascularization promotion, and immunosuppression (66). In melanoma, MMP-9 contributes to tumor cell invasion and primary tumor progression by remodeling the ECM. Neutrophils, fibroblasts, and endothelial cells are all capable of synthesizing and secreting MMP-9 (67).

MMP-9 is synthesized by both stromal and neoplastic cells in melanoma. The enhanced activity of this protease initiates the breakdown of specific ECM components, thereby promoting cellular motility by disrupting epithelial integrity. Additionally, MMP-9 activates key growth factors, including transforming TGF-β, VEGF, and TNF-α, thereby promoting tumor growth and angiogenesis (17, 68). Bogdan et al. (17) demonstrated positive MMP-9 expression in primary and metastatic tumor cells in 92% of cases (n=13 melanoma patients), with significantly higher expression in metastatic lesions than in primary tumors. These findings suggest that MMP-9 activation and overexpression promote melanoma invasion and metastasis. Furthermore, melanoma cells cultured *in vitro* exhibited stable MMP-9 activity that remained unaffected by culture duration, suggesting an intrinsic relationship between MMP-9 activity and melanoma invasiveness (69).

Subsequent research has demonstrated that MMP-9 promotes tumor cell invasion and metastasis by degrading the extracellular matrix, thereby enabling tumor cells to breach the basement membrane, infiltrate blood and lymphatic vessels, and form distant metastases (68, 70, 71). Moreover, MMP-9 promotes neovascularization in melanoma by modulating the secretion of VEGF and Angiostatin, consequently accelerating tumor growth and proliferation (72). *In vitro* and *in vivo* studies have demonstrated that interleukin-33 (IL-33) can significantly upregulate MMP-9 expression in macrophages within the melanoma-associated inflammatory microenvironment. The upregulated MMP-9 can cleave the NKG2D receptor on the surface of immune cells, impairing their ability to recognize cancer cell antigens. Simultaneously, it eliminates the expression of MHC class I molecules MICA/B on melanoma cells, thereby enabling tumor escape from T-cell recognition and promoting tumor survival. Treatment with the MMP-9 inhibitor SB-3CT restores T-cell-mediated tumor killing (26). Thus, MMP-9 may represent a novel immunotherapeutic target for melanoma treatment.

In summary, MMP-9 is a key regulator of melanoma development, invasion, and metastasis. It is produced by diverse cell types within the tumor microenvironment, such as tumor cells, fibroblasts, endothelial cells, and inflammatory cells, which enables it to influence multiple aspects of tumor progression. MMP-9 promotes melanoma malignancy through three principal mechanisms: degrading the extracellular matrix to facilitate invasion; activating growth factors such as TGF-β and VEGF to drive angiogenesis; and cleaving immune receptors such as NKG2D to suppress anti-tumor immunity. Preclinical studies confirm that inhibiting MMP-9 (e.g., with SB-3CT) can restore T-cell-mediated tumor killing and reverse aggressive phenotypes, highlighting its potential as a therapeutic target. However, current clinical evidence remains limited by small sample sizes and a lack of validated diagnostic or prognostic thresholds. Larger, multi-center prospective studies are needed to establish the clinical utility of MMP-9 as a biomarker and target in melanoma.

**Figure 6:**
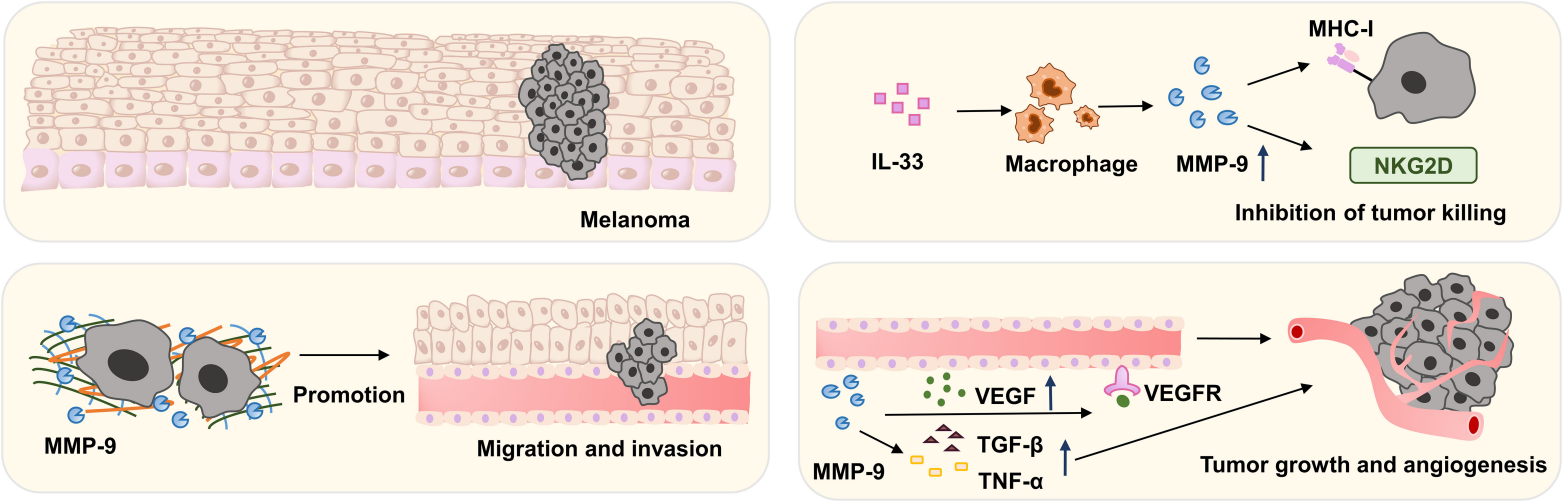
In melanoma, MMP-9 activates TGF-β, VEGF, and TNF-α, promoting tumor development and angiogenesis. MMP-9 also degrades extracellular matrix, enhancing tumor cell invasion and metastasis. In the inflammatory microenvironment of melanoma, IL-33 can significantly increase macrophage MMP-9 release. MMP-9 degrades immune cell NKG2D receptors and melanoma cell MHC class I molecules, weakening T cell antitumor action.

The involvement of MMP-9 in melanoma is shown in **Figure 6**.

**Figure 7:**
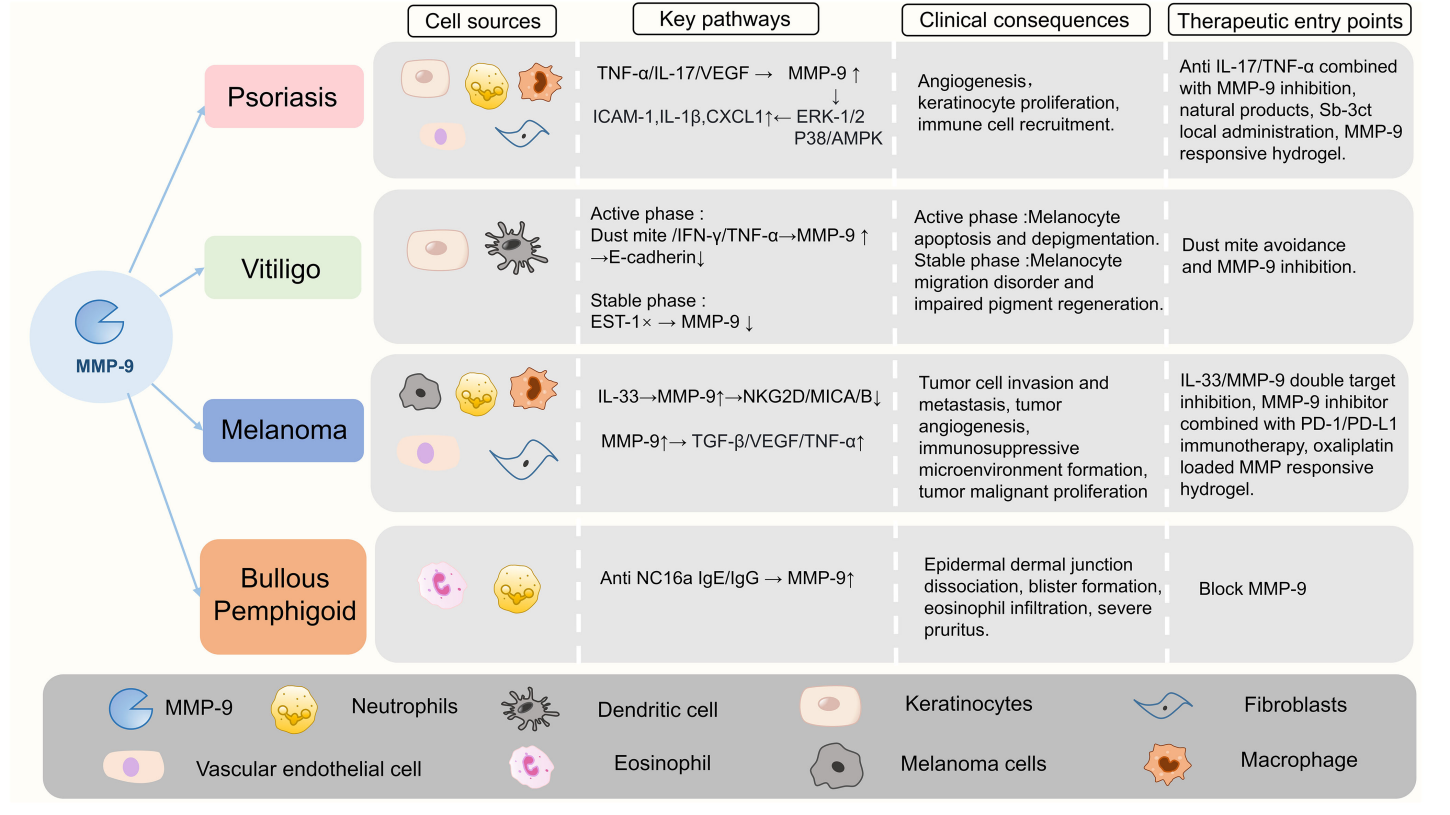
The shared and disease-specific roles of MMP-9 across psoriasis, vitiligo, BP, and melanoma.

The shared and disease-specific roles of MMP-9 in psoriasis, vitiligo, bullous pemphigoid (BP), and melanoma are shown in **Figure 7**.

The mechanisms of action and therapeutic significance of MMP-9 in immune-mediated skin diseases are shown in **Table 1**.

**Table 1:** The mechanism and therapeutic significance of MMP-9 in immune-mediated skin diseases.

Disease	The main cellular source of MMP-9	Principal upstream stimuli	Major downstream effects	Biomarker findings	Therapeutic implications
Psoriasis	Neutrophil	TNF-α	Angiogenesis, keratinocyte proliferation, immune cell recruitment.	AUC=0.77, serum and tissue expression levels ↑, positively correlated, with the severity of the disease.	Animal experiments have shown that inhibiting MMP-9 can alleviate psoriasis inflammation and improve skin lesions.
Vitiligo	Melanocyte, keratinocyte.	Ets-1, IFN-γ, TNF-α.	Immune cell recruitment, melanocyte damage.	Stable organizational expression level ↓, serum and tissue expression levels during the activity period ↑.	Undefined
Bullous Pemphigoid	Neutrophils, eosinophils.	Anti- Nc16A Ige/Nc16A Immune complex	Immune cell recruitment, epidermal-dermal separation.	Serum and tissue expression levels ↑, biological markers.	Animal and *in vitro* experiments have shown that blocking MMP-9 activity can reduce inflammatory infiltration and inhibit blister formation.
Melanoma	Neutrophils, fibroblasts, endothelial cells, tumor cells, macrophages.	IL-33	Angiogenesis, extracellular matrix degradation, immunosuppression.	Organizational expression level ↑.	Animal and *in vitro* experiments have shown that inhibiting MMP-9 activity can suppress the migration and invasion of melanoma cells.

## 4 Therapeutic strategies targeting MMP-9 in immune-mediated skin diseases

### 4.1 MMP-9-targeted therapeutic approaches

Matrix metalloproteinase-9, an MMP family protease, is a promising therapeutic target due to its function in several pathological processes, including tumor invasion and metastasis, angiogenesis, and inflammatory responses. Therapeutic strategies targeting MMP-9 have primarily focused on two main drug classes: small-molecule inhibitors and monoclonal antibodies.

Small-molecule inhibitors represented the primary focus of early research efforts. However, the first generation of broad-spectrum MMP inhibitors (Batimastat, Marimastat, and Cipemastat) faced significant challenges in clinical trials. The core issue was their lack of selectivity for MMP-9 within the MMP family, as broad-spectrum inhibition concurrently blocked the activity of physiologically essential MMPs.

Multiple Phase I studies showed that Batimastat successfully inhibited metastasis in malignant melanoma, but side effects such as fever, dyspnea, nausea, and peritonitis forced further studies to be halted. Marimastat, sharing a similar mechanism of action to Batimastat, advanced through preclinical studies but performed poorly in Phase II/III trials for metastatic cancers, inducing severe adverse reactions including pain, joint stiffness, muscle necrosis, and gastrointestinal ulcers. Furthermore, in some patients treated with Batimastat, MMP-1 inhibition impaired type I collagen interstitial remodeling, leading to severe tissue fibrosis (3). Subsequent inhibitors, such as Prinomastat, Tanomastat, and MMI 270 B, were discontinued in Phase III trials due to side effects—such as myelosuppression, gastrointestinal distress, and venous thrombosis—that outweighed their therapeutic benefits. The severe adverse effects associated with first-generation broad-spectrum MMP inhibitors greatly limited their clinical application, driving researchers to focus on developing highly selective MMP inhibitors.

Alternatively, monoclonal antibodies, which typically exhibit superior pharmacokinetic properties and reduced off-target toxicity, represent more promising MMP-9-targeted therapeutics. The efficacy of this approach has been preliminarily validated across multiple MMP targets. For example, monoclonal antibodies targeting MMP-14 (MT1-MMP) have demonstrated effective inhibition of tumor cell invasion in melanoma clinical trials (73). Building upon this precedent, MMP-9-specific monoclonal antibodies (Andecaliximab, AB0041, AB0046, GS-5745) have emerged as a research priority. These antibodies are currently undergoing rigorous preclinical evaluation to systematically assess their ex vivo efficacy and safety profiles, establishing the foundation for subsequent clinical investigation (74). Moreover, epigenetic modulation offers novel therapeutic avenues for inhibiting MMP-9. An *in vitro* study demonstrates that miR-143 overexpression in melanoma cells substantially downregulates MMP-9 at both transcriptional and translational levels, consequently attenuating metastatic capacity.

This finding introduces a promising therapeutic paradigm that uses RNA interference to inhibit MMP-9. These RNA-based approaches, including miR-143 mimetics and small interfering RNAs (siRNAs), exploit endogenous gene silencing machinery to achieve precise post-transcriptional knockdown of MMP-9 expression. This strategy offers enhanced target specificity with minimal off-target effects, representing a promising frontier in MMP-9-targeted therapeutics. However, prolonged MMP-9 inhibition may pose certain risks to normal cutaneous wound healing and barrier homeostasis. Balancing therapeutic efficacy with cutaneous protection remains a critical issue requiring further investigation.

### 4.2 MMP-9 responsive hydrogels

Substantial advances have been achieved in the application of MMP-9 responsive hydrogels for intradermal drug delivery. These hydrogels typically incorporate enzyme-sensitive peptide chains as crosslinks to enable rapid drug release. The hydrogel maintains its structural integrity in healthy skin, providing controlled, sustained drug release to reduce systemic exposure, whereas in MMP-9-overexpressing inflammatory lesions, it allows efficient cleavage of peptide crosslinks, thereby enabling rapid, on-demand local drug release. Currently, an MMP-9-responsive hydrogel-based smart drug delivery system has been successfully developed. It enables the targeted delivery of the JAK inhibitor tofacitinib to epidermal inflammatory sites, demonstrating potential for the treatment of inflammatory skin diseases (75, 76). Furthermore, this system offers the advantage of multifunctional drug-loading capacity, allowing for the co-encapsulation of multiple therapeutic agents to achieve synergistic effects. For example, by co-delivering cinnamyl alcohol (which inhibits endothelial cell ferroptosis) and exosomes derived from M2 macrophages, it can modulate remodeling of the epidermal immune microenvironment, thereby facilitating the transition of skin from a state of chronic inflammation to a proliferative repair phase (77, 78). In the treatment of melanoma, Liang S et al. (79) successfully developed an MMP-responsive injectable hydrogel. This formulation exhibits MMP-triggered degradation and sustained drug release, achieving complete release of the chemotherapeutic drug oxaliplatin and a model IgG within 3 days upon stimulation by MMP-2 and MMP-9, thereby inducing immunogenic cell death in tumor cells. *In vitro* and experimental studies demonstrated that the oxaliplatin and anti-PD-L1-loaded MMP-gel effectively inhibits both local and distant tumor growth, mitigates drug toxicity and side effects; and significantly prolongs survival.

In addition, MMP-9-responsive hydrogels have also achieved significant progress in the treatment of systemic and non-dermatological diseases, which holds important reference value for the development of MMP-9-responsive hydrogels for dermatological conditions. He et al. (80) found that MMP-9-related responsive mechanisms can enable precise intervention and dynamic monitoring of diabetic wounds. Wang et al. (81) demonstrated that such systems can remodel the balance of pro-/anti-inflammatory factors in the local wound microenvironment through MMP-9-mediated responses, effectively promoting the healing of diabetic wounds. In respiratory disease therapy, MMP-9-responsive nanogels loaded with catalase can achieve targeted regulation of inflammatory responses to treat eosinophilic asthma (82). Notably, the application of MMP-9 in oncology and vascular diseases has also garnered considerable attention. Li et al. (83) emphasized that the targeting and regulatory functions of MMP-9 are central to the efficacy of such nanomedicines in treating tumors and vascular disorders. Fu et al. (84) developed a targeted drug-delivery platform co-loaded with methotrexate and an MMP-9 inhibitor, which effectively downregulated angiogenesis-related molecules VEGF and CD31 in tumors and inhibited the Wnt/β-catenin signaling pathway through MMP-9 targeting, significantly enhancing tumor sensitivity to chemotherapy and opening new avenues for combination therapy based on MMP-9 targeting in oncology.

MMP-9-responsive hydrogels demonstrate considerable promise for optimizing therapeutic interventions in immune-related dermatoses. Their synergistic therapeutic efficacy stems from integrating targeted delivery properties with small-molecule inhibitors, potentially overcoming the limitations of conventional therapies. Future investigations should elucidate the local immunomodulatory effects to further advance their clinical application in immune-related dermatoses. Additionally, enhancing the hydrogel’s transdermal penetration and tissue residence time through nanocarrier modification will optimize delivery efficiency. Elucidating the regulatory mechanisms governing the local immune microenvironment will be pivotal for facilitating clinical translation.

### 4.3 Natural products and multi-target regulation

Natural compounds exert therapeutic effects by inhibiting MMP-9 transcription or blocking its enzymatic activity. In psoriasis treatment, geniposide ameliorate skin lesions by inhibiting MMP-9 expression and blocking the production of pro-inflammatory cytokines, including IL-23, IL-22, IL-17A, and TNF-α (85). Xijiao Dihuang Decoction has been demonstrated to alleviate psoriasis symptoms by downregulating MMP-9 levels at lesion sites, decreasing epidermal VEGFA expression, and interfering with pathological angiogenic processes, thereby reversing the increased neovascularization and vascular tortuosity observed in psoriasis models (86). *In vivo* animal experiments demonstrated that the Banzhilian formula could reduce inflammation and tissue destruction in psoriasis-like skin lesions using an imiquimod-induced psoriasis mouse model, while inhibiting the expression of Ki67 and inflammatory cytokines, including IL-17 and TNF-α, within the lesions. Furthermore, transcriptomic sequencing revealed that the anti-psoriatic effect of the Banzhilian formula was mediated by targeted modulation of the LCN2/MMP-9 signaling axis, providing robust mechanistic support for this traditional Chinese medicine formula and offering novel evidence for psoriasis treatment via MMP-9 inhibition (87).

In melanoma treatment, herbal monomers and their derivatives have demonstrated substantial therapeutic advantages. Research indicates that citrus coumarin can prevent melanoma cell migration and invasion in murine models by decreasing MMP-9 activity (88). Similarly, Atractylenolide I reduces MMP-9 secretion in melanoma cells and exerts anti-invasive effects by downregulating the JAK2/STAT3 signaling pathway (89). Galbanic acid (GBA) is a sesquiterpene coumarin compound abundantly found in Ferula species. Azad et al. evaluated the effects of GBA on melanoma cell invasion through invasion assays. Their findings revealed that after 24 hours of treatment, MMP-9 activity was reduced by 43.5%. Similarly, after 48 hours of GBA treatment, MMP-9 activity decreased to 29.5%. The findings indicate that GBA inhibits melanoma cell migration and invasion by suppressing MMP-9 activity, highlighting MMP-9’s potential as a therapeutic agent for melanoma treatment (90). Dihydroartemisinin, a compound extracted from Artemisia annua with recognized anticancer properties, has limited clinical application due to its poor solubility and toxicity profile. Kumar et al. (91) developed a dihydroartemisinin-loaded exosome formulation that reduced toxicity while enhancing oral bioavailability, effectively exerting anticancer activity and minimizing melanoma cell metastasis. *In vivo* animal experiments further confirmed that exosome-loaded dihydroartemisinin significantly reduced MMP-9 expression levels compared to dihydroartemisinin alone, thereby enhancing anticancer efficacy. These herbal monomers and their combinations exemplify the advantages of multi-target synergistic regulation, providing novel therapeutic insights for immune-related dermatological disorders.

A summary of the regulation of MMP‑9 by natural products for the treatment of immune‑mediated skin diseases is presented in **Table 2**.

**Table 2:** Summary of research on regulating MMP-9 with natural products for the treatment of immune-related skin diseases.

Natural products	Disease	Target of action	Level of evidence	References
Geniposide	Psoriasis	MMP-9, IL-23, IL-22, IL-17A, TNF-α.	Animal experiments and *in vitro* experiments	Liu, et al. (85)
Xijiao Dihuang decoction	Psoriasis	MMP-9, VEGFA.	Animal experiments	Guo et al. (86)
Banzhilian formula	Psoriasis	LCN2/MMP-9 signal axis, Ki67, IL-17, TNF-α.	Animal experiments	Xing et al. (87)
Citrus coumarin	Melanoma	MMP-9	Animal experiments	Hosseini et al. (88)
Atractylenolide I	Melanoma	JAK2/STAT3 signaling pathway, MMP-9.	*In vitro* experiment	Fu et al. (89)
Galbanic acid	Melanoma	MMP-9	*In vitro* experiment	Azad et al. (90)
Dihydroartemisinin	Melanoma	MMP-9	Animal experiments	Kumar et al. (91)

## 5 Discussion

MMP-9, as a core functional molecule within the MMP family, is extensively involved in the pathogenesis and progression of various inflammatory and neoplastic skin diseases, such as psoriasis, vitiligo, bullous pemphigoid, and melanoma, through multiple pathological mechanisms. These include modulating ERK1/2 signaling, regulating E-cadherin expression, contributing to eosinophil-dependent tissue damage, and mediating tumor immune evasion. Current fundamental and preclinical evidence consistently indicates that MMP-9 possesses dual translational value, serving both as a specific diagnostic biomarker and a precise therapeutic target, highlighting its significant potential for clinical exploration and application. To fully validate the therapeutic and biomarker potential of MMP-9, future research should focus on several key directions. First, conduct multi-center, large-sample prospective clinical studies. These should define standardized cutoff values for MMP-9 across different ethnic populations and disease subtypes, and verify its utility for monitoring treatment response and predicting long-term recurrence. Second, construct a multi-biomarker panel that integrates MMP-9 with disease-specific molecules (e.g., IL-17 in psoriasis, Ets-1 in vitiligo, and MICA/B in melanoma). This approach aims to enhance diagnostic specificity and prognostic accuracy beyond what single biomarkers can achieve. Third, standardize MMP-9 detection methods, such as serum enzyme-linked immunosorbent assay, tissue immunohistochemistry, or point-of-care testing, to ensure test results are reproducible and comparable in clinical settings. Fourth, explore the feasibility of using MMP-9 as a companion diagnostic biomarker for patient stratification in clinical trials of MMP-9-targeted agents. This could accelerate the translational process of novel therapies.

Traditional research models such as *in vitro* cell cultures and animal experiments have laid the foundational understanding of MMP-9’s functions in skin diseases but are inherently limited in simulating the complexity of the human skin microenvironment and the heterogeneity of clinical patients, making the application of multi-omics integration (transcriptomics, proteomics, epigenomics, and metabolomics) and single-cell sequencing technologies increasingly indispensable—single-cell RNA sequencing (scRNA-seq) enables the precise mapping of MMP-9-expressing cell subsets in lesional and non-lesional skin tissues, clarifying cellular heterogeneity and intercellular crosstalk, while multi-omics integration can systematically construct MMP-9-centered molecular regulatory networks, elucidating the “genotype-phenotype-function” correlation and overcoming the one-sidedness of single-omics studies. The clinical translation of MMP-9 inhibitors, a core direction of targeted therapy, relies on the design of ideal clinical trials featuring stratified enrollment based on disease subtype, activity stage, and baseline MMP-9 levels, prioritization of local targeted delivery strategies (e.g., MMP-9-responsive hydrogels) to reduce systemic toxicities, establishment of multi-dimensional efficacy evaluation systems combining clinical and molecular biological endpoints, and long-term safety monitoring to assess impacts on skin physiological functions such as wound healing and barrier integrity. Despite these prospects, current MMP-9-targeted therapy still faces critical limitations, including the lack of highly selective inhibitors (most existing agents are broad-spectrum, leading to off-target effects and systemic toxicities such as musculoskeletal discomfort), safety concerns associated with early broad-spectrum MMP inhibitors, and unclear clinical application scenarios and therapeutic response prediction indicators; addressing these challenges requires the development of selective inhibitors targeting MMP-9’s unique structural domains (e.g., fibronectin type II repeats, hemopexin-like domain), optimization of drug formulations and administration routes, combination with other targeted drugs to reduce dosage and toxicities, and large-scale prospective cohort studies to establish MMP-9-based multi-biomarker prediction models for personalized therapy. In essence, the in-depth exploration of MMP-9’s mechanisms through advanced technologies, the refinement of clinical trial design, and the resolution of current translational bottlenecks will collectively promote the clinical application of MMP-9-targeted strategies, providing new insights and therapeutic options for the precise management of immune-mediated skin diseases.
